# Supplementary material for: Analysis of Cultured Gut Microbiota Using MALDI-TOF MS in COVID-19 Patients from Serbia during the Predominance of the SARS-CoV-2 Omicron Variant
Source: Microorganisms. 2024 Aug 30;12(9):1800. doi: 10.3390/microorganisms12091800 (PMC11433956; doi:10.3390/microorganisms12091800)
Supplement: Supplementary file 1 [file microorganisms-12-01800-s001.zip › microorganisms-3141722-supplementary.pdf]

Table S1. Results of identification of bacterial and fungal isolates from fecal samples using MALDI-TOF MS.

**Phylum**	**Order**	**Genus**	**Species**	Fecal sample collected ≤ 5 days	Fecal sample collected 30-35 days	TOTAL
				No.isolates	No.isolates	No.isolates
**Firmicutes**	**Bacillales**	Bacillus	B. cereus	5	4	9
			B. pumiulus	5	8	13
			B. flexus	2	1	3
			B. mojavensis	4	4	8
			B. plantarum	5	5	10
			B. megaterium	1	3	4
			B. licheniformis	1	3	4
			B. subtilis	7	9	16
		Staphylococcus	S. hominis	3	1	4
			S. saprophyticus	2	1	3
		Sporosarcina	S. newyorkensis	3	3	6
			Sporosarcina luteola	3	2	5
		Lysinibacillus	L. fusiformis	3	1	4
		Psychrobacillus	P. psychrodurans	3	5	8
		Listeria	L. innocua	4	0	4
		Paenibacillus	P. amyloliticus	3	3	6
		Peribacillus	Peribacillus simplex	2	6	8
	**Lactobacillales**	Enterococcus	E.faecium	38	33	71
			E.faecalis	9	8	17
			E.durans	7	1	8
			E.hirae	3	2	5
			E. casseliflavus	0	1	1
			E.avium	1	1	2
		Leuconostoc	L. mesenteroides	0	2	2

		Lactococcus	L. lactis	1	5	6
		Lactobacilus	L. sakei	4	7	11
			L. plantarum	1	1	2
			L. reuteri	2	1	3
			L. paracasei	2	4	6
			Clostridium chauvoei	4	0	4
		Clostridium	C. novyi	0	3	3
			C. tertium	0	3	3
		Carnobacterium	C. maltaromanticum	0	2	2
			C. divergens	1	1	2
		Limosilactobacillus	L. mucosae	1	3	4
			L. antri	0	4	4
		Streptococcus	S. vestibularis	1	4	5
			S.lutetiensis	1	1	2
		Ligilactobacillus	L. saerimneri	0	2	2
		Filifactor	F. villosus	2	2	4
		Tissiarella	T.praeacuta	0	1	1
**Proteobacteria**	**Pseudomonales**	Pseudomonas	P. taetrolens	4	4	8
			P. fragi	4	4	8
			P. graminis	0	2	2
			P. jesenni	0	2	2
			P. lundensis	8	3	11
			P. proteolytica	1	1	2
			P. aeruginosa	1	3	4
			P. alcaliphila	1	1	2
	**Enterobacterales**	Klebsiella	K. oxytoca	4	0	4
			K. variicola	0	1	1
		Citrobacter	C. freundii	10	7	17
			C. braakii	11	7	18

			C. gillenii	0	4	4
		Enterobacter	E. cloacae	3	2	5
			E. hormaechei	1	1	2
			E. cowanii	2	1	3
			E. ludwigii	0	1	1
		Moellerella	M. wisconsensis	0	3	3
		Morganella	M. morganii	2	0	2
		Escherichia	E. coli	23	24	47
		Hafnia	H. alvei	20	20	40
		Raoultella	R. ornithinolytica	4	8	12
			R. planticola	1	3	4
		Yersinia	Yersinia rohdei	2	0	2
		Serratia	S. liquefaciens	1	0	1
		Proteus	P. mirabilis	1	0	1
	**Caulobacterales**	Brevundimonas	B. diminuta	2	0	2
	**Rhodocyclales**	Thauera	T. mechernichensis	2	2	4
**Actinomycetota**	**Micrococcales**	Arthrobacter	A.koreensis	2	4	6
			A. gandavensis	1	4	5
		Corynebacteriumm	C. mcginley	2	1	3
		Paenarthrobacter	P. aurescens	1	0	1
		Microbacterium	M. maritypicum	2	1	3
		Micrococcus	M.luteus	0	1	1
		Glutamicibacter	G. creatinolyticus	1	0	1
		Brevibacterium	B. casei	3	0	3
	**Streptosporangiales**	Nocardiopsis	N. alba	1	2	3
	**Mycobacteriales**	Rhodococcus	R. erythropolis	1	1	2
	**Actinomycetales**	Actinomyces	A. timonensis	4	4	8
			A. dentali	3	2	5
**Bacteroidetes**	**Bacteroidales**	Bacteroides	B. uniformis	1	0	1

	**Flavobacteriales**	Chryseobacterium	C. oncorhynchi	0	1	1
**Fungi**	**Saccharomycetales**	Candida	C. lusitanie	0	1	1
			C. krusei	1	1	2
			C. albicans	0	4	4
		Geotrichum	G. candidum	2	2	4
		Pichia	P. cactophila	2	3	5
	**Mucorales**	Mucor	M. circineloides	1	4	5
	**Sporidiobolales**	Rhodotorula	R. mucilaginosa	0	2	2
	**Eurotiales**	Aspergillus	A. niger	1	0	1
	**Tremellales**	Cryptococcus	C. neoformans	1	1	2
**TOTAL**				272	294	566
